# Neutralizing antibodies and safety of a COVID-19 vaccine against SARS-CoV-2 wild-type and Omicron variants in solid cancer patients

**DOI:** 10.1371/journal.pone.0310781

**Published:** 2024-11-07

**Authors:** Busyamas Chewaskulyong, Pattarapong Satjaritanun, Thanika Ketpueak, Thatthan Suksombooncharoen, Chaiyut Charoentum, Nuttaphoom Nuchpong, Apichat Tantraworasin

**Affiliations:** 1 Division of Medical Oncology, Department of Internal Medicine, Faculty of Medicine, Chiang Mai University, Chiang Mai, Thailand; 2 Medical Oncology Outpatient Clinic, Maharaj Nakorn Chiang Mai Hospital, Chiang Mai University, Chiang Mai, Thailand; 3 Department of Surgery, General Thoracic Unit, Faculty of Medicine, and Clinical Surgical Research Center, Chiang Mai University, Chiang Mai, Thailand; Universitas Syiah Kuala, INDONESIA

## Abstract

**Objective:**

The aim of this study was to assess the seroconversion rate and percent inhibition of neutralizing antibodies against the wild-type and Omicron variants of SARS-CoV-2 in patients with solid cancer who received two COVID-19 vaccine doses by comparing chemotherapy and nonchemotherapy groups.

**Methods:**

This prospective cohort study enrolled 115 cancer patients from Maharaj Nakorn Chiang Mai Hospital, Sriphat Medical Center, Faculty of Medicine, Chiang Mai University, and Chiang Mai Klaimor Hospital, Chiang Mai, Thailand, between August 2021 and February 2022, with data from 91 patients who received two COVID-19 vaccine doses analyzed. Participants received vaccines as part of their personal vaccination programs, including various mRNA and non-mRNA vaccine combinations. Blood samples were collected at baseline, on day 28, and at 6 months post-second dose to assess neutralizing antibodies. The primary outcome was the seroconversion rate against the wild-type and Omicron variants on day 28. Secondary outcomes included seroconversion at 6 months, factors associated with seroconversion, and safety.

**Results:**

Among the participants, 45% were receiving chemotherapy. On day 28, seroconversion rates were 77% and 62% for the wild-type and Omicron variants, respectively. Chemotherapy did not significantly affect seroconversion rates (p = 0.789 for wild type, p = 0.597 for Omicron). The vaccine type administered was positively correlated with seroconversion, with an adjusted odds ratio (95% confidence interval) of 25.86 (1.39–478.06) for the wild type and 17.38 (3.65–82.66) for the Omicron variant with the primary heterologous vaccine regimen. Grades 1 and 2 adverse events were observed in 34.0% and 19.7% of participants, respectively.

**Conclusions:**

Despite the lower seroconversion rate against the Omicron variant, no significant difference was observed between the chemotherapy and nonchemotherapy groups. COVID-19 vaccinations demonstrated good tolerability in this cohort. These findings highlight the importance of vaccine safety and immunogenicity in cancer patients and can inform tailored vaccination strategies for this vulnerable population.

## Introduction

COVID-19, an emerging infectious disease first reported in December 2019, is now a global pandemic caused by SARS-CoV-2. SARS-CoV-2 entry into host cells triggers an immune response, resulting in the release of inflammatory cytokines. This excessive inflammation drives high morbidity and mortality [1, 2]. In addition to wild-type viruses, novel variants significantly impact disease transmissibility, severity and the immune response [3]. Five major variants of concern (VOCs), including Alpha, Beta, Delta, Gamma and Omicron variants, have been reported [4].

Reports indicate that COVID-19 outcomes are worse in individuals with comorbidities [5], particularly in immunocompromised individuals such as cancer patients undergoing treatments, especially chemotherapy. Generally, chemotherapy not only affects quality of life but also dampens immunity, leading to increased susceptibility to and worse outcomes of infection [6, 7]. For COVID-19, cancer patients are more prone to severe infection outcomes, including increased rates of intensive care unit (ICU) admission, mechanical ventilation, prolonged hospital stays, and mortality [8, 9].

Studies on cancer patients have revealed decreased humoral immunity after infection and vaccination. Anti-spike antibodies and anti-nucleocapsid antibodies were once used as surrogate protective markers against SARS-CoV-2 infection in earlier studies [10]. Natural infection leads to reduced nucleocapsid immunoglobulin G (N-IgG) and spike immunoglobulin G (S-IgG) levels, especially after recent chemotherapy [11]. However, patients receiving immunotherapy presented increased antibody levels [12]. Similarly, mRNA-based vaccine studies have shown lower seroconversion rates (proportions of patients who develop detectable protective antibodies [13]) in cancer patients (90–94% after two vaccine doses) [14–16], with decreased neutralizing antibody levels against SARS-CoV-2 variants [17, 18]. However, humoral immunity declines over time, making a third booster dose necessary to maintain an adequate level of immunity [19]. Owing to the poor prognosis of some cancers, which is influenced by different factors, such as primary site, histological subtype, performance status, and stage, patients may have a shorter estimated life expectancy [20, 21], particularly those with advanced or metastatic disease [22]. Achieving a higher seroconversion rate even after two vaccine doses should be a concern because prompt protective immunity may be beneficial in these vulnerable patients to decrease susceptibility to SARS-CoV-2 infection and COVID-19-related hospitalization [23]. Data from noncancer populations revealed that heterologous prime-boosted vaccinations generated higher neutralizing antibody levels than did homologous vaccinations [24]. Further research is needed to obtain these data from cancer patients.

In this study, our objective was to assess the humoral-mediated immune response in terms of the seroconversion rate and percent inhibition of neutralizing antibodies against the wild-type and Omicron variants of SARS-CoV-2 in patients with solid cancer who received two COVID-19 vaccine doses, comparing chemotherapy and nonchemotherapy groups. Additionally, we aimed to investigate factors associated with antibody seroconversion on day 28 after completing vaccination and adverse events following immunization.

## Materials and methods

### Study design and participants

This observational prospective cohort study was designed to evaluate humoral immunogenicity in terms of surrogate neutralizing antibodies against the wild-type and XBB Omicron variants, as well as safety, in patients with solid cancer who received two doses of the CoronaVac vaccine. However, the protocol was adapted and amended later to allow different vaccine combinations because of vaccine shortages and uncertainties regarding vaccine management by the Thai government. Combinations of vaccines on different platforms, including mRNA vaccines with mRNA boosters, non-mRNA vaccines with non-mRNA boosters, and non-mRNA vaccines with mRNA boosters, were allowed by the Ministry of Public Health of Thailand. Notably, vaccine procurement and administration were not included in our study. This study included adult solid cancer patients aged 20 years or above with a confirmed diagnosis of cancer by histology or imaging at any stage and undergoing any treatments, including patients with complete remission of their disease within 1 year. The patients were required to have an estimated life expectancy of more than six months. All patients were followed at the medical oncology clinic at Maharaj Nakorn Chiang Mai Hospital, Sriphat Medical Center, Faculty of Medicine, Chiang Mai University, and Chiang Mai Klaimor Hospital (private hospital), Chiang Mai, Thailand. Exclusion criteria included having a previous diagnosis of SARS-CoV-2 infection based on RT‒PCR or antigen test kit (ATK) results in the past three months; having high-risk epidemiological factors within the past 14 days, for example, having close contact with an individual diagnosed with COVID-19 or visiting/living in an outbreak area; receiving prior COVID-19 vaccines; receiving other live attenuated vaccines in the past four weeks or inactivated and subunit vaccines in the past two weeks; having known allergies to any vaccine components; having signs and symptoms of active skin infection at the injection site; having HIV infection; receiving immunosuppressive drugs; receiving blood components within the past three months; being pregnant; having uncontrolled medical conditions; and having hematologic malignancies. The withdrawal criteria included inability to attend follow-up visits after receiving the vaccine and not completing the vaccination program. Patients who completed three vaccine doses and who had SARS-CoV-2 infection were also included. This study was approved by the Ethics Committee of the Faculty of Medicine, Chiang Mai University, with study code MED-2564-08326, approval number 348/2021. All participants received verbal and written information about the study and provided informed consent. The recruitment period for this study was from August 18, 2021, to February 28, 2022. This study was registered with the Thai Clinical Trials Registry (TCTR) ID: TCTR20230510001.

### Procedures and materials

#### Blood and data collection

A total of 157 patients with solid cancer were screened; 115 patients were enrolled in this study between August 2021 and February 2022. Data from 91 patients who had completed two vaccine doses were analyzed for neutralizing antibody levels. Demographic data were obtained, and 6 mL of blood was drawn for baseline analysis from participants on the day of signing the informed consent form. Each participant received a vaccine as part of their personal vaccination programs provided by the government or private hospitals, which included homologous and heterologous vaccine regimens.

Blood was obtained at 28 days and 6 months after the second vaccine dose. Third booster doses were allowed. Information on adverse events following immunization was collected during follow-up at the oncology clinic. Blood samples from participants were collected, centrifuged and stored as plasma samples in liquid nitrogen at the Division of Clinical Immunology, Department of Medical Technology, Faculty of Associated Medical Sciences, Chiang Mai University, Chiang Mai, Thailand, until further use for neutralizing antibody analysis and will be kept until five years after the completion of all analyses. Neutralizing antibody analysis was performed from March to September 2023.

#### Neutralizing antibody analysis

A surrogate virus neutralization assay kit (MAGLUMI, Shenzhen New Industries Biomedical Engineering Co., Ltd.) was used for the wild-type neutralizing antibody assay, and another neutralizing antibody kit (cPass™, GenScript, XBB variant spike protein) was used for the XBB variant neutralizing antibody assay. This method mimics the interaction between the host hACE2 receptor and the virus binding site via the recombinant SARS-CoV-2 receptor binding domain (RBD). This assay has shown 100% sensitivity and 100% specificity in clinical samples with confirmed SARS-CoV-2 virus neutralization titer (VNT50) values ≥ 20. Assay results included the antibody level in IU/mL units (wild type only), the percent inhibition, and whether the antibody was detected or undetected. Detected neutralizing antibody was defined as an antibody level greater than 121.6 IU/mL for the wild type and a percent inhibition greater than 30% for the Omicron variant. The XBB subvariant of the SARS-CoV-2 Omicron variant was chosen for analysis because it was the most common variant circulating in late 2022 [25].

#### Outcomes

The primary outcome was the seroconversion rate of neutralizing antibodies at 28 days after completing two vaccine doses against SARS-CoV-2 infection for both the wild-type and XBB Omicron variants. The secondary outcomes included the percent inhibition of neutralizing antibodies, seroconversion rates at 6 months, factors associated with seroconversion on day 28, and adverse events following immunization.

#### Statistical analysis

The required sample size was calculated to be 91 on the basis of previous seroconversion data. The data were analyzed per the protocol with the aim of reflecting data on vaccine efficacy. Descriptive data are reported as numbers and percentages, means and standard deviations (SDs), and medians and interquartile ranges. Chi-square tests and Fisher’s exact tests were used to compare the baseline characteristics between the chemotherapy and nonchemotherapy groups. The percent inhibition and seroconversion rates of neutralizing antibodies are reported as the means with 95% confidence intervals and were analyzed via repeated-measures mixed models across three time points (baseline, day 28, and month 6) and SARS-CoV-2 variants (wild-type and Omicron variants). Univariable logistic regression analysis was used to identify factors potentially associated with seroconversion. Factors with P < 0.1 were further investigated via multivariable logistic regression analysis. Statistical significance in each analysis was defined as P < 0.05. All the statistical analyses were performed via STATA/MP software version 17 (StataCorp LLC. College Station, TX, USA).

## Results

### Baseline characteristics

**Fig 1:**
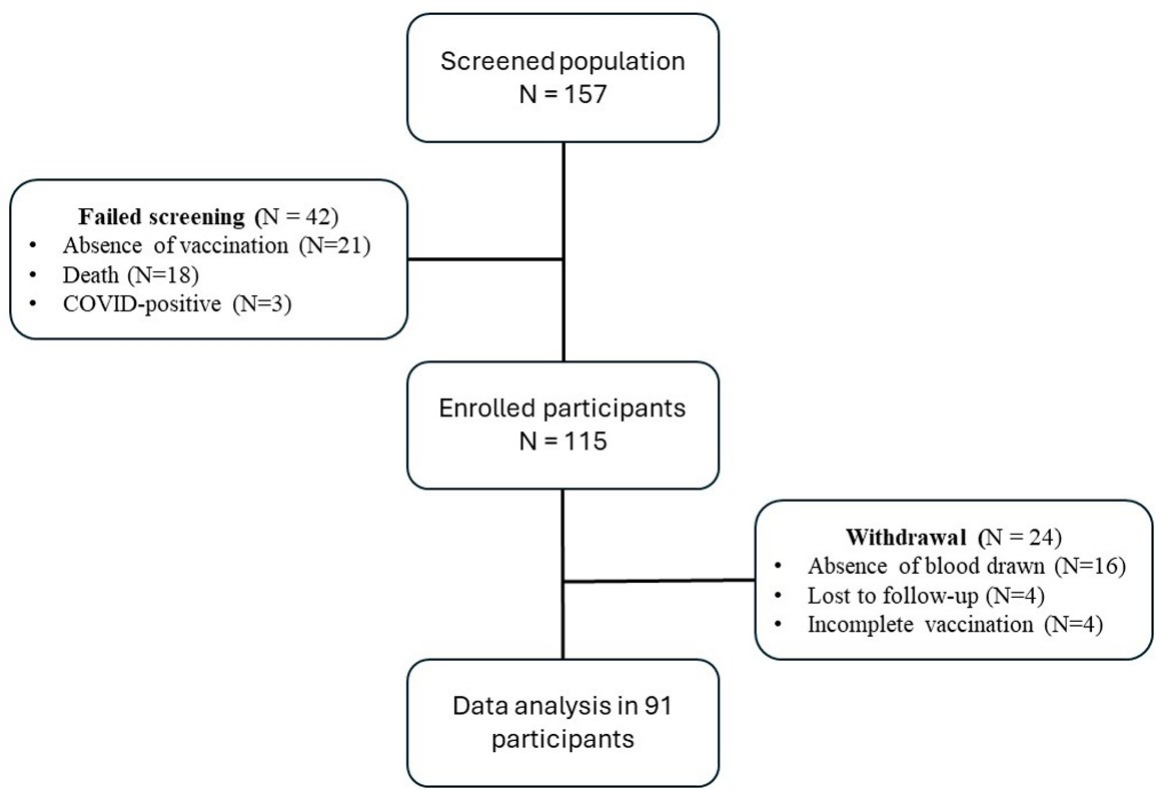
Study flow.

A total of 157 patients with solid cancer who planned to receive the COVID-19 vaccine were screened, among whom 115 were ultimately enrolled in this study. Data from 91 patients who had completed two vaccine doses were analyzed for neutralizing antibodies. The common reasons for failed enrollment were the absence of vaccination (n = 21/157, 13.3%) and death before completion of the vaccine course (n = 18/157, 11.4%), one of which was a COVID-19-related death. The failed screening and participant withdrawal data are depicted in Fig 1, which shows the study flow. chart The vaccines included homologous mRNA-based vaccines (mRNA+mRNA), homologous non-mRNA-based vaccines (non-mRNA+non-mRNA), and heterologous vaccines (non-mRNA+mRNA) in 41 (45%), 31 (34%), and 19 (21%) participants, respectively. Twenty-one of these individuals (23.0%) received a third-dose booster vaccine. All patients were then classified into chemotherapy (n = 41, 45%) and nonchemotherapy (n = 50, 55%) groups for further exploratory analysis, which were not prespecified subgroups.

Baseline demographic and disease characteristics are presented in Table 1. According to an observational-only study, some parameters between groups were not well balanced. In the chemotherapy group, male patients predominated (n = 26, 63.41%), whereas female patients predominated in the nonchemotherapy group (n = 31, 62.00%). The mean age and body mass index (BMI) were 60.76 years and 23.44 kg/m², respectively, for all participants. The most common primary cancers were gastrointestinal (GI) cancer (n = 21, 51.2%) in the chemotherapy group and breast cancer (n = 20, 40.0%) in the nonchemotherapy group. The baseline white blood cell, neutrophil, and lymphocyte counts tended to be lower, and hematologic adverse events during follow-up were more common in the chemotherapy group.

**Table 1:** Baseline demographic and disease characteristics.

Characteristics	All (n = 91)	Nonchemotherapy (n = 50)	Chemotherapy (n = 41)	*p* Value
Sex, n (%)				
Male	45 (49.45%)	19 (38.00%)	26 (63.41%)	0.016
Female	46 (50.55%)	31 (62.00%)	15 (36.59%)	
Age, years, mean ± SD	60.76 ± 11.78	59.48 ± 12.16	62.48 ± 11.62	0.234
BMI, mean ± SD	23.44 ± 4.48	23.21 ± 4.29 (n = 48)	23.50 ± 4.76	0.843
Smoking, n (%)	19 (20.88%)	7 (14.00%)	12 (29.27%)	0.075
Primary cancer, n (%)				
GI	32 (35.16%)	11 (22.00%)	21 (51.22%)	0.009
HBP	15 (16.58%)	8 (16.00%)	7 (17.07%)	
Breast	25 (27.47%)	20 (40.00%)	5 (12.20%)	
Prostate	5 (5.49%)	2 (4.00%)	3 (7.32%)	
Others	14 (15.38%)	9 (18.00%)	5 (12.20%)	
Metastatic disease, n (%)	44 (48.35%)	17 (34.00%)	27 (65.85%)	0.002
Comorbid, n (%)				
DM	17 (18.68%)	9 (18.00%)	8 (19.51%)	0.854
HT	34 (37.46%)	15 (30.00%)	19 (46.34%)	0.109
Other	40 (43.96%)	20 (40.00%)	20 (48.78%)	0.401
Vaccines, n (%)				
mRNA+mRNA	41 (45.05%)	21 (42.00%)	20 (48.78%)	0.688
Non-mRNA+mRNA	19 (20.88%)	12 (24.00%)	7 (17.07%)	
Non-mRNA+non-mRNA	31 (34.07%)	17 (34.00%)	14 (34.15%)	
Third vaccine booster	21 (23.08%)	12 (24.00%)	9 (21.95%)	0.817
WBC count, cells/mm^3^, median (IQR)	5985 (4400–7280)	6270 (4730–7540)	5040 (4160–6350)	0.085
Neutrophil count, cells/mm^3^, median (IQR)	3085 (2430–4190)	3560 (2520–4190)	2840 (2300–4560)	0.231
Lymphocyte count, cells/mm^3^, median (IQR)	1720 (1210–2270)	1870 (1370–2570)	1490 (950–1910)	0.057
Hematologic AE during follow up				
Leukopenia	15 (24.19%)	4 (12.9%)	11 (35.48%)	0.038
Neutropenia	5 (8.06%)	0	5 (16.13%)	0.020
Lymphopenia	20 (32.26%)	7 (22.58%)	13 (41.94%)	0.103

BMI = body mass index (mg/m^2^), GI = gastrointestinal cancer, HBP = hepatobiliary-pancreatic cancer, DM = diabetes mellitus, HT = hypertension, WBC = white blood cell, AE = adverse event

The vaccine regimens and their combinations are reported in Tables 2 and 3, respectively.

**Table 2:** Vaccine regimens.

	Company	Vaccine Platform
**Non-mRNA vaccines**		
AZD1222, ChAdOx1 nCoV-19	AstraZeneca	Replication-deficient chimpanzee adenoviral vector
CoronaVac, SinoVac	Sinovac Biotech	Whole inactivated virus
BBIBP-CorV, BIBP vaccine	Sinopharm	Whole inactivated virus
**mRNA vaccines**		
BTN162b2, Comirnaty	Pfizer–BioNTech	nucleoside-modified mRNA
mRNA-1273, Spikevax	Moderna	nucleoside-modified mRNA

**Table 3:** Vaccine combinations.

	All (91)	Nonchemotherapy (50)	Chemotherapy (41)
**mRNA + mRNA**	41	21	20
Pfizer+ Pfizer	21	8	13
Moderna+ Moderna	19	12	7
Others	1	1	0
**Non-mRNA+ mRNA**	19	12	7
AstraZeneca+Pfizer	18	11	7
Others	1	1	0
**Non-mRNA + non-mRNA**	31	17	14
Sinopharm + Sinopharm	17	12	5
CoronaVac + AstraZeneca	9	4	5
AstraZeneca + AstraZeneca	3	0	3
Others	2	1	1

### Neutralizing antibody analysis

#### Seroconversion rate

The baseline seroconversion rate before vaccination was 0% in all populations for both the wild-type and Omicron variants, confirming a seronegative status for SARS-CoV-2 in all participants. The seroconversion rate of the surrogate neutralizing antibody for the wild-type SARS-CoV-2 variant at 28 days after completing vaccination in all participants was 77% (95% CI: 67–85%). There was no significant difference in seroconversion rate between the chemotherapy and nonchemotherapy groups (76% vs. 78%, p = 0.789). In contrast, the seroconversion rates of the neutralizing antibody for the Omicron variant were 62% (95% CI, 51–72%), 59% (95% CI, 42–74%), and 64% (95% CI, 49–77%) in all participants and in the chemotherapy and nonchemotherapy groups, respectively, with no difference among the groups (p = 0.597). The seroconversion rate for the Omicron variant was lower than that for the wild type in both treatment groups (p = 0.008 for both the chemotherapy and nonchemotherapy groups). The seroconversion rates at 6 months did not differ from those on day 28 for either variant and are provided in Tables 4 and 5.

**Table 4:** Comparison between the chemotherapy and nonchemotherapy groups.

Neutralizing Antibody	Group		Baseline	Day 28	Month 6	*p* Value (Month 6 vs. Day 28)
Percent Inhibition (wild type)	Nonchemotherapy	N	48	50	45	
		Mean % (95% CI)	6.28 (4.87–7.69)	62.63 (53.13–72.12)	69.73 (59.95–79.51)	0.170
	Chemotherapy	N	41	41	35	
		Mean % (95% CI)	5.24 (4.01–6.47)	61.79 (50.79–72.78)	67.31 (56.17–78.44)	0.254
	*p* Value (chemo vs. nonchemo)			0.907	0.791	
	Total	N	89	91	80	
		Mean % (95% CI)	5.80 (4.86–6.74)	62.25 (55.19–69.30)	68.67 (61.48–75.86)	0.074
Percent Inhibition (Omicron variant)	Nonchemotherapy	N	48	50	45	
		Mean % (95% CI)	10.63 (8.72–12.54)	43.87 (36.59–51.16)	52.29 (42.99–61.60)	0.052
	Chemotherapy	N	41	41	35	
		Mean % (95% CI)	7.26 (5.45–9.07)	40.09 (32.19–47.99)	42.88 (32.99–52.77)	0.512
	*p* Value (chemo vs. nonchemo)			0.476	0.180	
	Total	N	89	91	80	
		Mean % (95% CI)	9.08 (7.73–10.42)	42.17 (36.90–47.44)	48.18 (41.45–54.90)	0.061
Seroconversion rate (wild type)	Nonchemotherapy	N	48	50	45	
		Mean % (95% CI)	0 (0–0.07)	0.78 (0.64–0.88)	0.80 (0.65–0.90)	0.778
	Chemotherapy	N	41	41	35	
		Mean % (95% CI)	0 (0–0.09)	0.76 (0.60–0.88)	0.83 (0.66–0.93)	0.236
	*p* Value (chemo vs. nonchemo)			0.789	0.743	
	Total	N	89	91	80	
		Mean % (95% CI)	0 (0–0.04)	0.77 (0.67–0.85)	0.81 (0.71–0.89)	0.365
Seroconversion rate (Omicron variant)	Nonchemotherapy	N	48	50	45	
		Mean % (95% CI)	0 (0–0.07)	0.64 (0.49–0.77)	0.71 (0.56–0.84)	0.316
	Chemotherapy	N	41	41	35	
		Mean % (95% CI)	0 (0–0.09)	0.59 (0.42–0.74)	0.60 (0.42–0.76)	0.875
	*p* Value (chemo vs. nonchemo)			0.597	0.311	
	Total	N	89	91	80	
		Mean % (95% CI)	0 (0–0.04)	0.62 (0.51–0.72)	0.66 (0.55–0.76)	0.420

**Table 5:** Comparison between wild-type and Omicron variants.

	Strain		Baseline	Day 28	Month 6
Percent inhibition (nonchemotherapy)	Wild type	N	48	50	45
		Mean % (95% CI)	6.28 (4.87–7.69)	62.63 (53.13–72.12)	69.73 (59.95–79.51)
	Omicron variant	N	48	50	45
		Mean % (95% CI)	10.63 (8.72–12.54)	43.87 (36.59–51.16)	52.29 (42.99–61.60)
	Wild type vs. Omicron, *p* Value			<0.001	<0.001
Percent inhibition (chemotherapy)	Wild type	N	41	41	35
		Mean % (95% CI)	5.24 (4.01–6.47)	61.79 (50.79–72.78)	67.31 (56.17–78.44)
	Omicron variant	N	41	41	35
		Mean % (95% CI)	7.26 (5.45–9.07)	40.09 (32.19–47.99)	42.88 (32.99–52.77)
	Wild type vs. Omicron, *p* Value			<0.001	<0.001
Seroconversion rate (nonchemotherapy)	Wild type	N	48	50	45
		Mean % (95% CI)	0 (0–0.07)	0.78 (0.64–0.88)	0.80 (0.65–0.90)
	Omicron variant	N	48	50	45
		Mean % (95% CI)	0 (0–0.07)	0.64 (0.49–0.77)	0.71 (0.56–0.84)
	Wild type vs. Omicron, *p* Value			0.008	0.103
Seroconversion rate (chemotherapy)	Wild type	N	41	41	35
		Mean % (95% CI)	0 (0–0.09)	0.76 (0.60–0.88)	0.83 (0.66–0.93)
	Omicron variant	N	41	41	35
		Mean % (95% CI)	0 (0–0.09)	0.59 (0.42–0.74)	0.60 (0.42–0.76)
	Wild type vs. Omicron, *p* Value			0.008	0.005

#### Percent inhibition

The percent inhibition was below the cutoff level for seroconversion in all participants at baseline. At 28 days after vaccination, the mean percent inhibition for the wild type was 62.25% (95% CI, 55.19–69.30), 61.79% (95% CI, 50.79–72.78), and 62.62% (95% CI, 53.13–72.12) for all participants and for the chemotherapy and nonchemotherapy groups, respectively (Table 3). There was no difference between the treatment groups (p = 0.907). For the Omicron variant, the mean percent inhibition was 42.17 (95% CI, 36.90–47.44), 40.09 (95% CI, 32.19–47.99), and 43.87 (95% CI, 36.59–51.16) for all participants and for the chemotherapy and nonchemotherapy groups, respectively. Again, there was no difference between the treatment groups (p = 0.476). However, the percent inhibition of the Omicron variant was lower than that of the wild type (p<0.001 in both treatment groups). The percent inhibition at 6 months is provided in Tables 4 and 5.

#### Factors associated with seroconversion

The univariable analysis of factors associated with the seroconversion of neutralizing antibodies for both the wild-type and Omicron variants is shown in Table 6. The results of the multivariable analysis presented in Table 7 revealed that the type of vaccine was the sole factor positively associated with seroconversion. For the wild-type SARS-CoV-2 variant, the adjusted odds ratios for the homologous mRNA vaccine (mRNA+mRNA) and heterologous vaccine (non-mRNA+mRNA) were 14.42 (95% CI 1.99–104.24, p = 0.008) and 25.86 (95% CI 1.39–478.06, p = 0.029), respectively. For the Omicron variant, the adjusted odds ratios were 8.90 (96% CI 2.93–26.94, p < 0.001) and 17.38 (95% CI 2.65–82.66, p < 0.001) for the homologous mRNA and heterologous vaccines, respectively. Diabetes mellitus (DM) was another potential factor associated with reduced seroconversion for the wild-type SARS-CoV-2 variant, with an adjusted odds ratio of 0.15 (95% CI: 0.02–1.02, p = 0.053), but the threshold for statistical significance was not met.

**Table 6:** Univariable analysis of explored factors for seroconversion for the wild-type and Omicron variants.

Factors	Wild Type			Omicron Variant		
	Seroconversion n (%)	OR (95% CI)	*p* Value	Seroconversion n (%)	OR (95% CI)	*p* Value
Sex						
male	36 (80.00) (n = 45)	1.39 (0.53–3.64)	0.670	29 (64.44%) (n = 46)	1.26 (0.54–2.92)	0.578
female	34 (73.91%) (n = 46)	ref	-	27 (58.70%) (n = 45)	ref	-
Age						
<65 yrs	47 (81.03%) (n = 58)	1.70 (0.63–4.57)	0.286	36 (62.07%) (n = 58)	1.22(0.52–2.88)	0.638
≥65 yrs	25 (71.43%) (n = 35)	ref	-	20 (57.14%) (n = 35)	ref	-
Smoking (yes)	16 (84.21%) (n = 19)	1.68 (0.44–6.44)	0.447	12 (63.16%) (n = 19)	1.14 (0.40–3.23)	0.801
BMI						
<18.5 (underweight)	9 (100%) (n = 9)	7.94 (0.42–148.19)	0.165	8 (88.89%) (n = 9)	3.73 (0.58–23.78)	0.163
18.5–22.9 (normal)	34 (79.07%) (n = 43)	1.51 (0.56–4.10)	0.410	24 (55.81%) (n = 43)	0.82 (0.34–1.98)	0.674
≥23.0 (overweight)	27 (71.05%) (n = 38)	ref	-	23 (60.53%) (n = 38)	ref	-
Primary cancer, n (%)						
GI vs. non-GI	25 (78.12%) (n = 32)	1.04 (0.37–2.91)	0.938	19 (59.38%) (n = 32)	0.92 (0.38–2.20)	0.857
HBP vs. non-HBP	13 (81.25%) (n = 16)	1.30 (0.33–5.07)	0.706	10 (62.50%) (n = 16)	1.09 (0.36–3.33)	0.867
Breast vs. nonbreast	18/26, 69.23% (n = 26)	0.53 (0.19–1.48)	0.229	14 (53.85%) (n = 26)	0.67 (0.27–1.69)	0.406
Prostate vs. nonprostate	3 (60.00%) (n = 5)	0.40 (0.06–2.61)	0.344	1 (20.00%) (n = 5)	0.14 (0.01–1.37)	0.093
Others vs. nonother	13 (92.86%) (n = 14)	4.40 (0.54–35.85)	0.166	13 (92.86%) (n = 14)	10.34 (1.28–82.92)	0.028
Metastasis	36 (81.82%) (n = 44)	1.72 (0.63–4.66)	0.286	28 (63.64%) (n = 44)	1.18 (0.50–2.76)	0.691
Active cancer treatment	51 (71.83%) (n = 71)	0.13 (0.01–1.07)	0.058	41 (57.75%) (n = 71)	0.45 (0.14–1.39)	0.167
Chemo vs. nonchemo	31 (75.61%) (n = 41)	0.87 (0.32–2.43)	0.788	24 (58.54%) (n = 41)	0.79 (0.34–1.85)	0.594
Targeted vs. nontargeted	11 (57.89%) (n = 19)	0.30 (0.10–0.90)	0.032	10 (52.63%) n = 19)	0.62 (0.22–1.74)	0.372
Hormonal vs. nonhormonal	8 (57.14%) (n = 14)	0.32 (0.09–1.07)	0.064	5 (35.71%) (n = 14)	0.28 (0.08–0.93)	0.038
No active cancer treatment	19 (95.00%) (n = 20)	ref	-	15 (75.00%) (n = 20)	ref	-
Comorbidity						
DM	11 (64.71%) (n = 17)	0.45 (0.14–1.43)	0.181	10 (58.82%) (n = 17)	0.90 (0.30–2.63)	0.848
HT	24 (68.57%) (n = 35)	0.46 (0.17–1.24)	0.128	18 (51.43%) (n = 35)	0.52 (0.22–1.25)	0.148
Other comorbid	30 (73.175%) (n = 41)	0.66 (0.25–1.76)	0.414	26 (63.41%) (n = 41)	1.21 (0.52–2.82)	0.654
Vaccine type						
mRNA+mRNA	40 (93.02%) (n = 43)	14.76 (4.06–53.65)	<0.001	32 (74.42%) (n = 43)	6.99 (2.54–19.19)	<0.001
non-mRNA+mRNA	19 (100.00%) (n = 19)	49.75 (2.76–895.38)	0.008	16 (84.21%) (n = 19)	11.66 (2.94–46.24)	<0.001
Non-mRNA+non-mRNA	14 (43.75%) (n = 32)	Reference		9 (28.12%) (n = 32)	Reference	
Leucopenia	11 (73.33%) (n = 15)	0.65 (0.16–2.52)	0.535	10 (66.67%) (n = 15)	1.35 (0.40–4.60)	0.624
Neutropenia	2 (40.00%) (n = 5)	0.14 (0.02–0.96)	0.046	2 (40.00%) (n = 5)	0.38 (0.06–2.51)	0.332
Lymphopenia	14 (70.00%) (n = 20)	0.46 (0.12–1.63)	0.234	12 (60.00%) (n = 20)	0.92 (0.31–2.74)	0.886

BMI = body mass index (mg/m^2^), GI = gastrointestinal cancer, HBP = hepatobiliary-pancreatic cancer, DM = diabetes mellitus, HT = hypertension

**Table 7:** Multivariate analysis of factors in the wild-type subgroup and Omicron variant subgroup.

Factors	Adjusted OR (95% CI)	*p* Value
**Wild-type subgroup**		
Neutropenia	0.23 (0.02–2.12)	0.199
Diabetes mellitus	0.15 (0.02–1.02)	0.053
Vaccine type		
Non-mRNA + non-mRNA	Ref	
mRNA+mRNA	14.42 (1.99–104.24)	0.008
Non-mRNA + mRNA	25.86 (1.39–478.06)	0.029
**Omicron variant subgroup**		
Cancer type		
Prostate	0.11 (0.01–1.34)	0.085
Other cancer	8.26 (0.81–84.08)	0.074
Vaccine type		
Non-mRNA + non-mRNA	Ref	
mRNA+mRNA	8.90 (2.93–26.94)	<0.001
Non-mRNA + mRNA	17.38 (3.65–82.66)	<0.001

### Safety

Grades 1 and 2 adverse events following immunization occurred in 34.0% and 19.7% of all participants, respectively, as shown in Table 8. The most common side effect was pain at the injection site, followed by fever and fatigue. There were no serious adverse events leading to emergency department visits or hospitalizations.

**Table 8:** Adverse events.

Adverse events	All Participants (n = 91)		Nonchemotherapy (n = 50)		Chemotherapy (n = 41)	
	Grade 1 n (%)	Grade 2 n (%)	Grade 1 n (%)	Grade 2 n (%)	Grade 1 n (%)	Grade 2 n (%)
Any	31 (34.0%)	18 (19.7%)	22 (44.0%)	8 (16.0%)	9 (21.9%)	10 (24.3%)
Pain at injection site	14 (15.3%)	10 (10.9%)	11 (22.0%)	3 (6.0%)	3 (7.3%)	7 (17.0%)
Fever	8 (8.7%)	5 (5.4%)	5 (10.0%)	2 (4.0%)	3 (7.3%)	3 (7.3%)
Fatigue	4 (4.3%)	3 (3.2%)	4 (8.0%)	3 (6.0%)	0	0
Malaise	3 (3.2%)	0	1 (2.0%)	0	2 (4.8%)	0
Diarrhea	1 (1.0%)	1 (1.0%)	1 (2.0%)	0	1 (2.4%)	1 (2.4%)

### Clinical correlation of seroconversion and SARS-CoV-2 infection

Four participants (n = 4/91, 4.39%) were confirmed to have SARS-CoV-2 infection after completing vaccinations; all of them tested negative for seroconversion for both the wild-type and Omicron variants. The timing periods of infection ranged from 1 to 11 months after the second vaccine dose. Among them, one patient suffered from severe COVID-19 pneumonia, required mechanical ventilation, and experienced multiorgan failure, leading to death. In contrast, none of the seropositive participants were diagnosed with SARS-CoV-2 infection.

## Discussion

Our study was initially designed during the era of vaccine shortages in Thailand and worldwide. According to government policy at that time, the procurement of COVID-19 vaccines was disorganized, and access to vaccines relied on personal efforts. Therefore, vaccine combinations with different platforms were expected to be heterogeneous unintentionally.

This study analyzed a real-world cohort of patients with solid cancer, which has a very high mortality rate. Apart from suffering from their cancer, these patients are also vulnerable to SARS-CoV-2 infection. Eleven percent of the participants (n = 18) died due to cancer-related problems, including one death from COVID-19 pneumonia, and 3 percent (n = 4) were confirmed to be positive for SARS-CoV-2 infection before completing vaccination or blood analysis. Given the grim prognosis of cancer, a higher rate of seroconversion to protective antibodies against SARS-CoV-2 infection after two vaccine doses is still a concern, although a third booster dose is crucial for maintaining immunity.

Compared with analysis of anti-RBD or anti-S antibodies, analysis of neutralizing antibodies can serve as a more predictive tool for assessing protection against SARS-CoV-2 infection, as these antibodies can bind to and neutralize the virus, aiding in viral control and clearance [26]. Anti-RBD and anti-S antibody levels are poor predictors of immunity for the wild-type and novel variants, as increasing levels of anti-RBD do not necessarily imply the presence of neutralizing antibodies [27]. Data from previous reports may overestimate vaccine efficacy, highlighting the importance of determining vaccine efficacy on the basis of neutralizing antibodies. Surrogate neutralization assays offer an alternative effective method that does not require a biosafety level 3 laboratory and exhibits high sensitivity (95–100%) and specificity (99.93%) [28]. The test still performs acceptably after validation for the Omicron variant [29]. Our study revealed a lower seroconversion rate of surrogate neutralizing antibodies (77% and 62% for the wild-type and Omicron variants, respectively) than did previous reports on the seroconversion of anti-RBD and anti-S antibodies in patients with solid cancers (90–94%) [14, 30] and in the healthy population (97–99%) [31]. This result is consistent with a study on the pseudovirus neutralization assay in cancer patients, where detectable neutralization antibodies for the wild-type virus were found in 75–85% of the group receiving mRNA-based vaccines [18].

Multiple variants of SARS-CoV-2 have evolved from the wild-type virus since the beginning of the outbreak, mostly due to different mutations under selective pressure at the S and RBD regions; furthermore, these variants also influence disease transmissibility, severity and immunity after infection and vaccination [3]. Currently, there are five VOCs, including Alpha, Beta, Delta, Gamma and Omicron variants [4]. The XBB subvariant of Omicron was the most prevalent variant worldwide in 2022, the study time period [25]. The lower seroconversion of the Omicron variant (XBB) in this study is consistent with other reports in both cancer and healthy populations [32, 33]. Most COVID-19 vaccines were developed before the era of ongoing novel VOCs, leading to challenging problems with vaccine effectiveness [34]. In the general population, risk reductions for incidence and mortality are predominantly associated with the Alpha variant but are diluted by the Delta and Omicron variants; these effects can be overcome by booster doses [35]. In the capture study, seroconversion of neutralizing antibodies via live virus microneutralization assays was found to be positive in 83, 61, 53, and 54% of patients with cancer for the wild-type, Alpha, Beta, and Delta variants, respectively [17]. Another study of lung cancer patients receiving mRNA vaccines revealed more than 50-fold decreased levels of neutralizing antibodies to the Omicron variant compared with those to wild-type SARS-CoV-2 in a live virus neutralization assay [32]. This finding is in concordance with that of our study. Additionally, the mean percent inhibition decreased from 62.25% for the wild type to 42.17% for the Omicron variant. Thus, these findings suggest a limited efficacy of vaccines against VOCs.

Active cancer treatments, including chemotherapy, targeted therapy, endocrine therapy, and immunotherapy, have controversial outcomes with respect to seroconversion. Among these agents, chemotherapy is considered an immunosuppressive agent that might cause leukopenia and lead to infection. This concept has elicited interest in the potential detrimental effects of chemotherapy on vaccine efficacy. Some studies have shown a reduced humoral immune response in chemotherapy treatment groups [36–38]. In a large cohort study of U.S. veterans, patients receiving chemotherapy within 3 months before vaccination had the lowest vaccine effectiveness, which was 57% (95% CI: 23 to 90%), when compared with the endocrine therapy and no systemic therapy groups (76%, 95% CI: 50 to 91%; and 85%, 95% CI: 29 to 100%, respectively) [39], whereas some studies have revealed no mitigatory effect of chemotherapy on humoral immunity in the context of COVID-19 vaccines [40, 41]. Our study revealed a decreasing trend but no statistically significant difference in either the seroconversion rate or percent inhibition in patients receiving chemotherapy three months prior to or during the vaccination period in terms of neutralization antibodies for both the wild-type and Omicron variants, regardless of leukopenia, neutropenia, or lymphopenia. However, the overall effects of systemic treatments for cancer and vaccination on SARS-CoV-2 infection outcomes remain unclear and inconsistent [42, 43].

Differences in vaccine platforms result in unequal immunogenicity. Compared with non-mRNA vaccines, mRNA-based vaccines generate greater amounts of RBD antibodies and neutralizing antibodies in cancer patients [44, 45]. The exploratory analysis of factors associated with seroconversion from our study suggested that only the type of vaccine combination was related. Compared with homologous non-mRNA vaccines, the primary heterologous vaccine combination yielded the highest seroconversion outcome, with an adjusted odds ratio (ORR) of 25.86 (95% CI 1.39–478.06, p = 0.029), followed by homologous mRNA vaccines, with an adjusted ORR of 14.42 (95% CI 1.99–104.24, p = 0.008), for the wild type. This result was similar for the Omicron variant, with adjusted odds ratios of 17.38 (95% CI 2.65–82.66, p < 0.001) and 8.90 (96% CI 2.93–26.94, p < 0.001) for heterologous and homologous mRNA vaccines, respectively. Undoubtedly, booster doses after completing two COVID-19 vaccine doses generate higher and longer-lasting neutralizing antibody levels [19, 46, 47]; consequently, a third booster vaccine is essential and should be regarded as the standard of care. In a large, matched control cancer cohort study in Singapore, the clinical benefit of vaccine for preventing severe disease was even greater with a four-dose mRNA-based vaccine regimen [48]. To date, the clinical guidance for COVID-19 vaccination approved by the U.S> Centers for Disease Control and Prevention (CDC) recommends three doses of mRNA vaccines for immunocompromised individuals [49].

However, this study provides a novel report on the more robust immunogenicity of heterologous primary vaccines in patients with cancers, which is coherent with reports on healthy populations [50] and a third heterologous prime-boost vaccine [24]. The potential mechanisms include the reorientation of B-cell responses toward neutralizing sites of expressed epitopes encoded by mRNA vaccines [51] and the accompanying cellular and humoral responses on different vaccine platforms [52]. Therefore, the heterologous vaccine strategy should be encouraged as the second booster primary vaccine, particularly in populations that tend to have lower seroconversion rates.

Diabetes Mellitus (DM) was another potential factor contributing to a weaker immune response in patients with wild-type SARS-CoV-2 infection, with an adjusted odds ratio of 0.153 (95% CI: 0.023–1.022, p = 0.053); however, the threshold for statistical significance was not met. DM is well known for its immunosuppressive state. Some systematic reviews in the general population reported the inferiority of the immune response to the COVID-19 vaccine in patients with DM, particularly those with poor glycemic control [53, 54]. DM and glycemic control in patients with cancer should be evaluated further regarding adverse correlations with neutralizing antibodies.

Regarding safety concerns, vaccinations were well tolerated in both the chemotherapy and nonchemotherapy groups. All the participants experienced minor reactions, such as pain at the injection site, fever, and fatigue, which were self-limiting or alleviated by over-the-counter drugs. None of the participants needed to seek medical attention or required hospitalization.

The strength of our study lies in providing data on COVID-19 vaccine safety and immunogenicity, specifically in terms of the seroconversion of surrogate neutralizing antibodies, in solid cancer patients with and without active cancer treatments, especially chemotherapy for advanced or metastatic disease. However, our study has several limitations. The population size was small due to the high mortality rate of cancer patients, incomplete vaccination, and lack of blood samples from some participants. Second, missing information on third, booster vaccines during the follow-up period resulted in an inaccurate analysis of neutralizing antibodies at 6 months. Hence, the longevity of the neutralizing antibodies could not be determined in our study. In addition, this study did not include an analysis of the cellular immunity and memory function of the adaptive immune response.

## Conclusions

Our study revealed that in solid cancer patients, COVID-19 vaccination leads to substantial immune responses, with seroconversion rates of 77% for the wild type and 62% for the Omicron variant. Heterologous vaccines were more effective, and chemotherapy did not significantly affect the seroconversion of neutralizing antibodies. The adverse events were mostly mild, confirming the safety of the vaccines. Further studies on cell-mediated immunity, current circulating variants and clinical benefits of COVID-19 vaccines beyond increasing neutralizing antibody levels in cancer patients will provide additional valuable information.

## Supporting information

S1 Dataset (XLSX)
